# What to expect when girls are expecting: psychosocial support challenges and opportunities in the context and aftermath of teenage pregnancy in Kenya

**DOI:** 10.1186/s12978-022-01544-1

**Published:** 2022-12-21

**Authors:** Chi-Chi Undie, Harriet Birungi

**Affiliations:** Population Council, P. O. Box 17643-00500, Nairobi, Kenya

**Keywords:** Teenage pregnancy, Early pregnancy, Psychosocial support, Psychosocial support for parents, Adolescent mental health, Kenya

## Abstract

**Background:**

An understanding of the psychosocial support challenges and opportunities in the context of teenage pregnancy is important for developing appropriate interventions for pregnant and parenting girls. This qualitative study was conducted in Homa Bay County, Kenya, to examine the experience of teenage pregnancy and the resultant psychosocial support needs from the perspectives of both pregnant/parenting girls and their own parents, who are typically expected to provide various forms of support.

**Methods:**

The study used a descriptive case study design, drawing on counseling case notes documented by psychologists who held counseling sessions with 20 pregnant or parenting girls and 6 of their parents as part of a psychosocial support intervention. The counseling case notes formed a qualitative data set, which was analyzed thematically.

**Results:**

Emerging master themes were related to pregnant and parenting girls’ experience of sexual violence and adverse childbirth outcomes; psychological trauma confronted by girls and their parents alike; parental need for support in communicating with pregnant/parenting girls about sex and sexuality; and the availability of family support as a resource for teenage mothers.

**Conclusion:**

Pregnant and parenting teenage girls require a range of psychosocial support responses that recognize the realities of sexual violence and other challenges in the lives of the girls themselves, as well as in the lives of their parents and caregivers. While parents and other caregivers can serve as an important resource for supporting affected girls, they often need assistance as well, in order to support pregnant/parenting girls effectively. These realities need to be taken into account to maximize the effectiveness of health and development programs for pregnant and parenting girls. Furthermore, emerging themes from actual counseling sessions with affected girls and parents can provide important insights into the potential psychosocial support needs of the broader population of pregnant and parenting girls.

## Plain language summary

This study looked into the lives of pregnant and parenting girls in Kenya by examining case notes that were written up by psychologists during the counseling sessions they held with 20 different teenage girls who were pregnant, or already had children, and 6 of their parents. The case notes provided an understanding of the kind of support pregnant and parenting girls need, including mental health care to deal with the trauma of sexual violence (and other issues) that some had experienced. It also helped put a spotlight on the traumas that parents of affected girls face after a teenage pregnancy occurs. These issues need to be addressed in order to ensure that health and development professionals who work with pregnant and parenting girls are as helpful to them as possible.

## Background

Despite the worrisome prevalence of teenage pregnancy in Africa—with nearly 1 in 5 adolescent/teenage girls becoming pregnant in this region [1]—the evidence base on the psychosocial support concerns of affected girls within this context is surprisingly thin. The current literature on the experiences of teenage mothers (including psychosocial support/mental health issues) is characterized by ‘few studies’ [2], with evidence on best practices around psychosocial support for pregnant and parenting girls described as being ‘limited’ [3]. Yet, early pregnancy is associated with tremendous psychosocial effects [2, 4, 5], which are likely to be exacerbated in the low-resource settings that many African countries represent.

Recent review studies support these findings, highlighting the dearth of evidence on this subject in Africa, in particular. For instance, an integrative review of adolescent girls’ experiences of transitioning to motherhood [6] included only one Africa-based study. More recently, a systematic review of psychosocial interventions designed for pregnant and parenting girls [3] found no eligible studies in low- and middle-income countries, and called for a focus on the psychosocial needs of this population to better target their mental health outcomes.

A number of studies have begun to respond to this call, noting that the psychosocial support needs of pregnant and parenting teenage girls in Kenya, for instance, revolve around issues such as economic support [7], mental health concerns [8–10] and stigma [10, 11]. These important psychosocial support issues emerged from conventional datasets focusing on pregnant/parenting girls and other related populations. However, data derived from actual psychosocial support contexts are yet to inform the emerging body of literature. Yet, such data have the potential to deepen our understanding of a complex issue requiring urgent solutions.

To address this gap, this paper draws on the voices and experiences of pregnant/parenting girls (and, by extension, their parents) in the context of home-based counseling sessions held in Homa Bay County, Kenya. Examining affected girls’ lives in this unique context provides an opportunity to gain deeper insight into their worlds, and to pick up on critical psychosocial support challenges and needs that have yet to make their way into the literature. Such evidence is critical for informing the design of effective health and development programs and policies for pregnant and parenting girls.

## Methods

### Study context, sample, design

This study draws on a qualitative, secondary data set composed of counseling case notes recorded by trained psychologists from November to December 2019. The psychologists were engaged to support a data collection team carrying out a household survey with girls and their parents conducted in three sub-counties within Homa Bay County, Kenya, namely, Homa Bay, Ndhiwa, and Rangwe [12]. Nationally in Kenya, Homa Bay County has the second highest childbearing levels among girls ages 15–19 [13]: A third of girls in this age range are pregnant and/or parenting, compared to 18% of their peers, nationwide.

Under the household survey, pregnant and parenting girls were offered home-based counseling and referrals for psychosocial support, in recognition of the response needs associated with these populations. A field team conducted the household survey, and psychologists doubled up as interviewers when they were not engaged in counseling sessions. A total of 20 pregnant/parenting girls requested counseling by a psychologist during or after their interviews. Although counseling support was intended solely for girls, parental demand for similar support resulted in about a third of the girls’ parents (6 out of the 20 cases) being counseled as well. Pregnant/parenting girls who responded in the affirmative when asked whether they would like to speak to someone further regarding any of their psychosocial issues were included in the current study, along with any parents of such girls who proactively asked to speak to someone. Pregnant/parenting girls and parents who requested to speak to someone were not eligible for inclusion in the study if they could not speak to the psychologist privately. Three psychologists attached to the household survey team documented counseling case notes for all pregnant/parenting girls (along with the parents concerned) that asked for counseling. The present study is qualitative in nature and based on the narratives contained in these case notes.

Standard validation techniques employed in qualitative research were applied to gauge the alignment of this study’s findings with reality. These techniques involved devoting attention to the qualitative constructs of confirmability (comparable to the notion of objectivity in quantitative research), dependability (comparable to ‘reliability’ in quantitative methods), and credibility (comparable to internal validity in quantitative methods) [14]. The confirmability of the data in this study was enhanced by preserving an audit trail of the data collection, processing, and reduction that led to the study’s conclusions, providing evidence of the grounding of these conclusions in the actual data. The dependability of the data was enhanced by minimizing the number of psychologists recording the case notes (one per sub-county), and through holding check-in meetings with the psychologists to discuss emerging counseling and case note recording issues. To assess the credibility of the data, areas of uncertainty and negative evidence were deliberately sought out within the data set, and alternative explanations were considered and weighed against the existing data prior to arriving at conclusions.

The study used a descriptive case study design. This type of case study design is employed ‘to describe an intervention or phenomenon and the real-life context in which it occurred’ [15]. Homa Bay County served as a single, holistic case within which the phenomenon of teenage pregnancy was explored, along with the psychosocial support issues associated with it, and the context in which these occurred. The descriptive case study drew on the case notes emanating from counseling sessions with pregnant/parenting girls at the household level. Where a parent of a pregnant/parenting girl also requested counseling at the household level, the resultant case notes were merged with those of the index pregnant/parenting girl to gain a deeper understanding of her overall context. This led to a total of 20 case notes (representing 20 pregnant/parenting girls). Of these, 6 case notes included descriptions of counseling sessions with the parent of the girl concerned.

### Data collection and analysis

The psychologists on the data collection team hand-recorded their ‘counseling case notes’ (i.e., a record of their management of each girl that requested counseling, along with descriptions of the counseling encounter and the context in which it occurred). Their documentation of the cases involved a two-pronged process reminiscent of fieldnote documentation: initial ‘jottings’ about the counseling encounter (in order not to forget important issues raised by the client, and to be able to follow up on them during the counseling session), followed by the expansion of the notes later in the day [16, 17]. Once the case notes were fully written up, they were processed in Microsoft Word and anonymized before being shared electronically with the corresponding author.

Data from the case notes were analyzed manually and thematically – a process that entailed reading through a first case note in its entirety and documenting a list of tentative themes identified at ‘descriptive’ and ‘conceptual’ levels [18]: straightforward descriptions of specific experiences were noted, in addition to more complex concepts that required deeper reflection and piecing together.

Themes were either confirmed or disconfirmed by the first author after reading subsequent case notes in their entirety and finding that they either re-emerged or did not. New tentative themes were also added to the list as the reading of subsequent case notes ensued, and they were subjected to the same process of confirming/disconfirmation by comparing them to other case notes in the data set. The final list of themes was produced via multiple readings of all case notes to be sure nothing was overlooked or erroneously labeled as a theme without evidence of multiple tellings across counseling cases.

### Ethical considerations

Several levels of ethical approval were obtained prior to conducting the overall study from which this paper’s data were derived, including from the Ethics and Research Committee of the Kenyatta National Hospital-University of Nairobi in Nairobi, Kenya, and from the New York-based Institutional Review Board of the Population Council. In addition to the consent and assent processes required when interviewing minors, the study also took into account the potential trauma that interview questions on early pregnancy could pose for pregnant and parenting girls. Three psychologists were therefore incorporated into the data collection team to provide first line psychosocial support (in the form of immediate counseling) to girls who asked to speak to a professional. In addition to providing first line counseling, the psychologists were trained to provide referrals for further care to those who had received counseling. The project established a relationship in advance with the Kenya Association of Professional Counselors in the neighboring County of Kisumu to attend to any acute cases referred.

## Results

Five overarching themes emerged from the analysis, revolving around sexual violence, adverse childbirth outcomes, psychological trauma (of girls and their parents alike), parents’ need for support, and the availability of family support for pregnant/parenting girls. These themes are unpacked in the sub-sections below.

### Sexual violence

Sexual violence turned out to be the most prominent theme across the majority of the case notes, with many girls indicating that they had gotten pregnant as a result of rape. Indeed, only 5 out of the 20 pregnant/parenting girls who received home-based counseling became pregnant as a result of consensual sex. Oftentimes, incidents of sexual violence occurred in the context of girls’ ‘boyfriend-girlfriend’ relationships. Other perpetrators that emerged from the case notes included relatives, friends, and strangers.

Pregnant girls also struggled with disclosure, due to the notion that their experience of rape was their fault, and that support and sympathy would not be readily available.

I was interviewing this girl, and when we got to the section on sexual experiences, she started crying. I stopped the interview and asked her if she would like to talk to me as I assured her of total confidentiality. She trusted me and she opened up that she is pregnant, and she has not told anyone, including her mom. She is fearing her mom will kick her out of the house and she has no idea what to do. She said her boyfriend, who is in [secondary school], forced her to have sex when she didn’t want to: “My boyfriend was escorting me one evening and he started ‘touching-touching’ me. I told him to stop, and he refused to. Before I knew it, he had pulled me in the bush and had sex with me. I tried to scream, but he covered my mouth. After he finished, he warned me never to tell anyone. I have not gotten my period for 3 months now, and when I went to the hospital, I was told I am pregnant. I’m so scared [about] what my parents will say. *Counseling case notes, 13-year-old girl*

Her mother left her and her brothers behind under the care of their paternal step uncle, while their father travelled to Nairobi. Two days later, the said uncle raped her. She said she screamed and her … brother, who was then 12 years old, raised alarm and neighbors came to their rescue. She remembers how she bled profusely and was admitted to the hospital for two weeks. The uncle ran away and disappeared. He was being sought by police but could not be traced. A few days later, his body was found in the forest. He had hung himself. During [the] burial, the family agreed the matter was to be kept a secret, while her step grandmother threatened she would kill them all for causing her son to commit suicide. She never told anyone about this story, not even her own mother. *Counseling case notes, 15-year-old girl*

The girl … didn’t want to continue with the interview. I stopped the interview and asked her if there was anything that she would like to share, and she mentioned that her uncle visited them and found her parents had gone for a burial and took advantage of her. The girl mentioned that if she tells her parents, they will blame her: ‘’Kindly don’t share this with them. This is the person I trusted most, but he betrayed me. He pulled me into the house, and I tried to scream, and he warned me that if I continued, he would kill me. He had sex with me and warned me never to tell anyone. This has made me feel so bad … I hate him and I don’t like seeing him or even his wife.” *Counseling case notes, 13-year-old girl*

### Adverse childbirth outcomes

Some teenage mothers returning to school (or trying to) were grappling with raising children with health issues, or with the loss of their children, for example. The case notes served as a reminder of the intersecting challenges faced by teenage mothers who did not come away with the expected birth outcome of a healthy child:

She is the second born in a family of 5 children. Her childhood development was hampered by [a] delay in walking till age three. She started school at age six and but at some point she repeated primary 7 three times. During that period, she was raped by a stranger, and she conceived. She continued [going to school] until delivery. It was a pre-term baby delivered at six months. … Her baby is stunted in growth – seemingly has hydrocephalus. He is 1 year and six months old, but cannot walk. *Counseling case notes, 16-year-old girl*

She was referred by [a data collection team member] following the loss of her 3-day old infant (had breathing problems from birth). The interviewer reported to me that she was teary during their interview session … [Her] baby died after she was discharged from the hospital. *Counseling case notes, 15-year-old girl*

### Psychological trauma

#### Girls’ experiences

During their counseling sessions, pregnant/parenting girls showed evidence of psychological trauma as a result of their pregnancies (and other associated concerns), whether the latter were rape-related or not. All psychologists noted the deep traumatization of the girls concerned, with one specifying that, while the first line of counseling provided was important and helpful, many of the girls actually required long-term psychotherapy.

The sexual debut of many girls occurred in the context of sexual violence. Several girls found it challenging to disclose their horrifying experiences to anyone, and had not spoken about them until they were asked if they wanted to speak to someone during data collection for the wider study.

The circumstances described above resulted in a wide range of psychological problems and mental health issues described by pregnant and parenting girls, including depression, fear, suicidal ideation, insomnia, reduced self-esteem, and hopelessness, as outlined below:

(The girl started crying). I gave her time and I asked her if she was ready to continue. This was after doing a bit of counselling and she was okay… She said, “[A boy I met on the road while running an errand] locked me up for two days and threatened that if I dare screamed, he would kill me. I didn’t know his parents had travelled and he knew he was all alone, and nobody would know what was happening. He had sex with me, which was the worst experience that I have ever had. I cried, but he was not ready to let me go. He forced me. After the second day, he let me go, but he warned me never to tell anyone. The experience was very scary, and I didn’t tell my mom or anyone. I thought she would beat me. I used to cry every day[.] … After one month, I noticed some changes and I started throwing up and getting sick. My mother took me to the hospital, and I was pregnant. This broke me into pieces, and I didn’t have a way forward. I wanted to commit suicide[.] *Counseling case notes, 15-year-old girl*

The girl was very innocent, and she hadn’t started her period yet. … She remembers this day when there was a function at his place [a family friend and peer]. “[E]veryone, including my family, attended. … It was a bit dark [when it was time to go home], and I was scared. He offered to escort me and since he was like a brother, I didn’t mind. … All of the sudden, he started removing my clothes and I had no idea what was going on. I told him to stop, but he refused. I got so confused and before I knew it, he started ‘doing bad manners to me’ [having sex with me]. I screamed and I cried, but he covered my mouth. Once he was done, he left me and warned me never to tell anyone. I was very scared, and I didn’t sleep for 2 weeks. I had nightmares. After a few months, I started seeing my stomach [growing] big. I had no idea what was happening.” *Counseling case notes, age of girl not recorded*

Her sleep is not smooth: “I see demons and sometimes wake up screaming loudly,” she said. *Counseling case notes, 17-year-old girl*

I then did a … [counseling] session with … [the girl] who was very traumatized and had threatened suicide. This was the most difficult session because, at the middle of the session, the girl broke down. She said her mom and siblings have not been talking to her and they have no idea what happened. “I was raped,’’ said the girl. She said nobody ever asked her [what happened]. I gave her time to calm down and later on she explained to me that her boyfriend, who was in [secondary school], forced her to have sex when she was on her way home from church. “He convinced me to stop by his house and he locked the door and raped me. I tried to scream, but he covered my mouth. I didn’t know I got pregnant until I missed my period. *Counseling case notes, 15-year-old girl*

This particular girl was forced to have sex by her boyfriend, and she got pregnant. She was very traumatized, and she feels useless. “I feel as if there is no need of living,” the girl said. She said … when she visited [her boyfriend] as she used to do, he pulled her into the bedroom and started to undress her. She tried to shout, but he covered her mouth. “I have never had sex before, and this left me pregnant.” *Counseling case notes, 14-year-old girl*

#### Parents’ experiences

The case notes analysis also surfaced the psychological trauma confronted by the parents of pregnant and parenting girls. This trauma manifested in the forms of sadness, despair, bitterness, and anger, for instance, and led some parents to act out against their pregnant/parenting daughters, including by being harsh (verbally and physically), belittling pregnant/parenting girls before their siblings, lashing out, and simply ignoring or ostracizing their daughters. This often led to situations of ‘ping-pong’ trauma within affected girls’ households, as such girls inadvertently inflicted trauma on their parents as a consequence of their pregnancy/parenthood status, while parents, in turn, unknowingly triggered further trauma in their daughters when they left their own trauma unattended to. The quotes below illustrate the complex psychological terrain navigated by parents of pregnant and parenting girls:

Her mom was so bitter after realizing she was pregnant that she didn’t say a word to her. … She told me she is a single mother, and her daughter has really disappointed her: “Can’t they see how I am struggling?’’ she said, as she broke into tears. She told me her [elder] daughter … also got pregnant in school, and she had to struggle with the baby and her. She thought her younger sister would have learnt from her [experience]. She said she doesn’t want to see her, or even find out the person responsible for the pregnancy: ‘’If she wants, let her leave.’’… After a long counseling [session], the mom was satisfied, and she was ready to support her daughter. She said talking about the issue had helped her and she feels better now. *Counseling case notes, mother of 15-year-old girl*

I counseled [the pregnant girl] and I explained to her the importance of disclosing to her parents and asked her she if was ready to tell her mother herself. She agreed and she told her mom, who was very devastated. I had a deep counseling session with her mom, and she told her daughter she should have reported to her as soon as [the rape] happened. She would have helped by taking her to the hospital. She said she had no idea how she would break the news to the girl’s dad. *Counseling case notes, mother of 13-year-old girl*

The father … would occasionally speak badly to her [making remarks] such as, “You! You’re still seated at home, baby-sitting, while your classmates are in school, studying.” … I had a [counseling] session with [him], which was fruitful and productive. He admitted that indeed he was disappointed in his daughter, since she was the most quiet and humble of his children, and finding out that she became pregnant because a boy bought her *mandazis* [Kenyan doughnuts] worth 20 shillings was very disappointing, to say the least. He admitted that, in his anger, he had spoken harsh words to his daughter. *Counseling case notes, father of 17-year-old girl*

### Parents’ need for support

A recurrent theme among parents of pregnant and parenting girls was their need for external support in talking to their daughters about sex and sexuality. After participating in the quantitative survey under the wider study, parents of pregnant/parenting girls commonly asked for someone to speak to their affected daughters about these issues, admitting their difficulty in doing so themselves.

The following narratives depict this communication challenge faced by parents of pregnant/parenting girls:

[O]ne of the Head Teachers … had two teenage girls, and after interviewing the girl who was sampled in the study, he requested me to counsel his daughters: “You know, these things affect us as well. We are also parents, despite being leaders at school.” He said that this is one of the reasons he was advocating for girls to remain in school as long as they want (even if they are pregnant). He said some Head Teachers may never understand until this menace of teenage pregnancy strikes them. I had a very successful session with [his] two open girls, who said they had experienced a lot of challenges with their peers at school. … The parents came to thank me and told me this is what they need in the community: Someone to talk to the girls. The dad [Head Teacher] said it’s hard, at times, to talk about sex with their girls. *Counseling case notes, father of a 14-year-old teenage girl*

On finishing up the interview [with this household head/parent], she asked, “What can we do for these girls of ours who still get pregnant even after we to try meet their needs?” … I [empathized with] her frustrations, while helping her identify her strengths as a parent [e.g., by] meeting her daughter’s needs, but at the same time, pointed out to her some of the gaps that most parents experience during parenting – like [in the area of] sex education/talks … which, she agreed, is always a tough topic. *Counseling case notes, mother of 16-year-old girl*

A mother … requested the data collectors to help counsel her … daughter who gave birth and went back to school, [along with] her eldest daughter, who was equally intelligent, but whom she feared would be influenced. This mother of six and widow feared that her young daughter might have not learned from her previous experience, and may be taken advantage of again, [even though her mother] was struggling to provide for them with her meager income (she sold roasted groundnuts for a living). *Counseling case notes, 13-year-old girl*

The fact that all parents who received counseling responded positively to this service (even when they had not requested for it, but were drawn into counseling by the proactive psychologists) is a further indication of the sheer need that parents have for support in order to properly support pregnant/parenting girls in turn.

### Availability of family support

Despite the displeasure of most parents about their daughters’ teenage pregnancies, the vast majority expressed willingness to support their daughters’ return to school, or were already doing so. Parental support was demonstrated in other ways, as well, including by showing kindness and unconditional love toward their daughters, despite their disappointment, or by providing emotional support.

“I wanted to commit suicide, but my mom encouraged me and walked the entire journey with me. I would have been dead by now. Later on, I gave birth, and this was very traumatizing, and I felt the world had come to an end. I thank God for the support I got from my parents. And after a few months, I told them I wanted to go back to school. They were ready to take me back to school, and that is why I am in Class 8 now, waiting for the national exam results to be out, and I’m expecting good results.” She talked of how her parents are struggling financially to keep her in school. *Counseling case notes, 15-year-old girl*

She was not planning to tell her mother [about her pregnancy], but after helping her come into terms [with it] and understand the importance of disclosure, she explained to her mom [what happened]. She thought her mom, who is very [strict], would kick her out of her house, but her mom was so shocked and lost for words. But she promised to support her in going back to school, since she was a very intelligent girl. *Counseling case notes, 14-year-old girl*

At the end of the counselling session, which was very long, we involved the parents, who were very supportive, especially [in regard to] making sure she goes through school. *Counseling case notes, 15-year-old-girl*

Notably, grandmothers emerged several times as avid supporters of their pregnant/parenting granddaughters. Grandmothers’ homes appeared to be places of succor for affected girls.

[After having a baby], she relocated to her maternal grandmother’s home, where she currently goes to school. *Counseling case notes, 16-year-old girl*

The mother was supportive and urged her to … be at peace. Thereafter, she was taken to grandmother’s place, where she delivered[.] *Counseling case notes, 15-year-old girl*

Her paternal grandmother loves her and her baby, so she will possibly take care of the baby while she will be at school. *Counseling case notes, 16-year-old girl*

## Discussion

This study is associated with some limitations: In particular, its core findings emanated from the narratives of a small, select group of pregnant and parenting girls who requested counseling support when it was offered, and from the accounts of an even smaller group composed of affected girls’ parents. We therefore craft our discussion of the findings with great care, and with the important caveat that our conclusions are not driven by a concern for ‘representativeness’ in the quantitative sense. They are, rather, propelled by the conceptual issues emerging from the data. As Ulin and colleagues [19] remind us, ‘Our goal [with qualitative inquiry] is to produce data that are conceptually, not statistically, representative of people in a specific context’. It is noteworthy, nonetheless, that the key findings from this study are in concurrence with results from several population-based studies, which are also discussed in this section. Findings from this study provide a portrait of existing psychosocial support challenges and opportunities in the context of teenage pregnancy, based on the lived experiences of some affected girls and parents in Kenya. This portraiture could serve as a useful starting point for developing psychosocial support interventions for pregnant and parenting girls in low- and middle-income countries where such interventions are rare [3].

Sexual violence, for instance, turned out to be the most prominent theme emerging across the majority of the case notes, with 15 out of the 20 girls in the sample indicating that they had gotten pregnant as a result of rape. The high prevalence of sexual violence among pregnant/parenting girls is reflective of findings from an earlier, population-based study in the same context, which showed that over a third (35%) of ever pregnant 12- to 16-year-olds reported getting pregnant as a result of rape or coercion [20].

Oftentimes, sexual violence occurred in the context of girls’ ‘boyfriend-girlfriend’ relationships, supporting findings from a population-based study in the same sites which indicated that about 90% of teenage mothers reported getting pregnant by their boyfriends, with a considerable proportion of these boyfriends (37%) being their fellow students at the time [21]. Other perpetrators that emerged from the case notes included relatives, friends, and strangers. Pregnant girls also struggled with disclosing their experience of sexual violence.

The realities of girls in this study who experienced sexual violence mirror those of their peers nationwide: A nationally-representative survey in Kenya [22] demonstrated that 43% of 13- to 17-year-old girls who experienced an incident of sexual violence in the past 12 months did not tell anyone about their experience. Screening interventions to support sexual violence disclosure have been observed to be feasible and effective in Kenyan settings [23] and could potentially be embedded into psychosocial response efforts for pregnant and parenting girls. Additionally, perpetrators were often the girls’ schoolmate boyfriends or peers, and pregnant/parenting survivors were reticent about disclosing sexual violence. These findings draw our attention to the need to incorporate boys into the portrait of teenage pregnancy. Related to this is the critical need to embed sexuality and relationships education (which would include sensitization to certain concepts such as gender-based violence, consent, self-management, kindness, and relationship skills [24], for example) in the discourse and programming around teenage pregnancy. Such education is clearly required early on in the lives of girls and boys within and outside of school contexts. While the Kenyan government has demonstrated support for sexuality education, it has privileged abstinence-only approaches [25], which, in addition to being incomprehensive, overlook the realities of sexual violence (and other grave, sexuality-related issues) occurring as early as primary school age.

The need for sexuality and relationships education is all the more important, given parents’ recurrent request in the present study for external support in communicating with their children about sex and sexuality. This parent-child communication challenge plausibly stems from cultural prescriptions in some Kenyan sub-cultures, which hold that conversations about sex and sexuality contravene notions of ‘respect’ within parent-child relationships [26]. Consequently, parent-child communication around these subjects is often only prompted by negatively-perceived events, including early pregnancy [27]. Sexuality and relationships education efforts could, therefore, be designed to support parents in this regard, and to complement related endeavors within and outside schools.

The study’s findings demonstrate that mental health concerns are not the preserve of pregnant/parenting girls alone. Parents of girls who had experienced pregnancy sometimes faced psychological trauma of their own – an issue that has received much less attention in the literature. Intersections between adolescent and parental trauma also requires a response and further investigation in the context of teenage pregnancy, given evidence of their co-occurrence in this study. While this intersection could potentially have a cyclical effect, prolonging recovery for both girls and their parents, it also holds potential for whole-family healing, if properly addressed. Although evidence of this intersection emerged only once in the present study (in a case where a pregnant girl and her mother alike were physically abused by her father in response to her teenage pregnancy), intersections between violence against girls and violence against women in the context of teenage pregnancy are also worth exploring further, given the likelihood that this is occurring much more often than this qualitative study was able to capture.

Adolescent mothers face higher risks of adverse birth outcomes than older mothers [28]. As seen in this study, this reality can place extra pressure on girls whose babies face health challenges (or whose babies do not survive), coupled with the stigma of being a young mother of school-going age, and the prevalent experience of rape-related pregnancy. Adverse birth outcomes and their psychosocial effect on teenage mothers are rarely considered, and should become a part of response interventions for this population.

It is also important to highlight existing family support for pregnant and parenting girls as a major theme that emerged in this study. Childcare is a major barrier to parenting girls’ advancement [21]. Nonetheless, as the study findings suggest, parents, grandparents, and other relatives can serve as an important childcare resource to support the future endeavors of pregnant and parenting girls. Grandmothers, in particular, emerged from the case notes analysis as relatives who offered a safe space and ‘guaranteed’ support for pregnant/parenting girls. These family resources should be part and parcel of psychosocial responses in low-resource settings.

Overall, the findings from this study are quite similar to results from research around the psychosocial issues of teenage mothers in non-African contexts. Interpersonal violence, mental health challenges, and economic issues are commonly experienced among pregnant and parenting girls in other settings as well [4, 5, 29], and incorporating the parents and extended family of teenage mothers into support interventions is considered important and effective [4]. Parent-teen communication about sexuality issues is also noted as being challenging in other settings [30]. However, conduct disorders among pregnant/parenting teens did not emerge in the current study, but were highlighted as relevant in the broader literature [5]. Its absence from the present study could be due to the small sample size (which may not have allowed for certain issues to be captured), or could be associated with sociocultural factors in Kenya that might moderate conduct problems (such as oppositional defiant disorder) in households with pregnant/parenting teenage girls. The broader literature also extends the framework of psychosocial support, going beyond pregnant/parenting girls and their parents alone, to include the children of teenage mothers, who are noted to require psychosocial support as well [4, 5]. Future psychosocial studies and interventions that give attention to the children of teenage mothers would be valuable for strengthening needed responses.

## Conclusion

Pregnant and parenting teenage girls require a range of psychosocial support responses that recognize the realities of sexual violence and other challenges in the lives of the girls themselves, as well as in the lives of their parents and caregivers. While parents and other caregivers can serve as an important resource for supporting affected girls, they often need assistance as well, in order to support pregnant/parenting girls effectively. These realities need to be taken into account to maximize the effectiveness of health and development programs for pregnant and parenting girls. Furthermore, emerging themes from actual counseling sessions with affected girls and parents can provide important insights into the potential psychosocial support needs of the broader population of pregnant and parenting girls.

## Data Availability

The dataset analyzed during the current study is available from the corresponding author on reasonable request.
